# Structural Stability of AM/AMPS/AMB Terpolymers Under Simulated Extreme Oilfield Conditions

**DOI:** 10.3390/polym18111393

**Published:** 2026-06-04

**Authors:** Peng Xue, Jingxing Wang, Junwei Fang, Qingjie Ma, Zhi Kang, Linghui Xi, Xiumin Dong, Yi Zhang, Zuguo Yang, Long He

**Affiliations:** 1 Northwest Petroleum Branch, Sinopec, Urumqi 830011, China; xuep.xbsj@sinopec.com (P.X.);; 2 Key Laboratory of Enhanced Oil Recovery for Fractured-Vuggy Reservoirs, Sinopec, Urumqi 830011, China; 3 College of Chemistry and Chemical Engineering, Central South University, Changsha 410083, China

**Keywords:** computational design, high-temperature and high-salinity gel blocker, molecular dynamics simulation, oilfield chemistry, AM/AMPS/AMB copolymer

## Abstract

Water management in high-temperature and high-salinity reservoirs remains a critical challenge for oilfield operations, with conventional polymer gel systems exhibiting insufficient thermal stability and salt tolerance under extreme conditions. Here, we establish an integrated computational–experimental platform combining density functional theory (DFT) and molecular dynamics (MD) simulations to rationally design a novel AM/AMPS/AMB (Acrylamide/2-acrylamido-2-methylpropanesulfonic acid/sodium 3-acrylamido-3-methylbutanoate) terpolymer gel plugging agent tailored for the Tahe Oilfield (140 °C, Ca^2+^/Mg^2+^ 10,000 mg L^−1^). Density functional theory (DFT) calculations of fourteen functional monomers identified AMB as the optimal candidate, achieving further hydrogen bond interactions that stabilize the crosslinked architecture under extreme conditions. This computational pre-screening reduced experimental iterations by over 60% and significantly shortened development cycles compared to conventional trial-and-error approaches. Experimentally, the optimized terpolymer exhibited a 40% increase in storage modulus (150 Pa) relative to AM/AMPS binary systems, 25% improvement in thermal stability (residual carbon at 300 °C), and plugging efficiency exceeding 92% in core flooding tests.

## 1. Introduction

Excessive water production during oilfield flooding operations remains a persistent challenge [1,2,3], particularly in high-temperature and high-salinity reservoirs where premature water breakthrough severely compromises oil recovery and economic viability [4,5]. Water plugging technology, which selectively seals high-permeability zones to reduce the oil–water flow ratio, has emerged as an essential solution [6,7]. Specifically designed water plugging agents have been required to seal high permeability layers, reducing the oil–water flow ratio, mitigating water breakthrough, and enhancing oil recovery according to different local conditions [8,9,10]. However, conventional water shutoff agents—primarily inorganic materials or simple polymer systems—exhibit inadequate stability and performance degradation under extreme reservoir conditions [11,12], necessitating the development of more robust polymer-based alternatives [13].

Acrylamide (AM) represents the most widely utilized monomer for oilfield water plugging applications due to its excellent water solubility and polymerization characteristics [14]. While AM-based polymers undergo rapid hydrolysis and molecular chain scission when exposed to elevated temperatures and high brine concentrations, leading to progressive viscosity loss and diminished plugging efficiency [15,16,17]. Copolymerization with 2-acrylamido-2-methylpropanesulfonic acid (AMPS) partially addresses these limitations by introducing sulfonate groups that enhance salt resistance and thermal stability [18,19,20]. Nevertheless, AM/AMPS binary systems still fall short of the performance thresholds required for the most challenging environments, such as the Tahe Oilfield (140 °C, Ca^2+^/Mg^2+^ 10,000 mg L^−1^), prompting exploration of ternary systems incorporating specialized functional monomers [21].

The introduction of functional monomers—characterized by specific structural elements that impart enhanced hydrophobicity, chemical stability, or acid/base resistance—offers a promising pathway for further property optimization [22,23], enhancing the stability of copolymers under harsh environmental conditions [24]. Recently, ternary copolymer systems based on AM, AMPS, and functional monomers that containing fluorine, nitrogen, or phosphorus to integrate additional benefits such as corrosion and aging resistance [25], have gained significant attention in recent studies [26]. Nevertheless, identifying optimal monomer combinations for specific reservoir conditions through traditional experimental screening remains labor-intensive, time-consuming, and inherently uncertain [27,28]. This experimental bottleneck has created an urgent need for predictive design tools that can efficiently navigate the vast chemical space of potential copolymer formulations.

Computational chemistry and molecular simulation have emerged as powerful alternatives to conventional trial-and-error approaches for oilfield water plugging agents [29,30]. Density functional theory (DFT) enables precise evaluation of electronic-level interactions between monomer units, while molecular dynamics (MD) simulations reveal conformational behavior, thermodynamic properties, and network stability under reservoir-relevant conditions [31,32,33]. By establishing quantitative structure-property relationships [34], these computational tools can guide rational copolymer design and dramatically accelerate development timelines [35,36], offering a theoretical foundation and practical support for advancing oilfield water plugging technology [37,38].

In this study, we establish an integrated “computational screening–synthetic validation” platform to design and optimize AM/AMPS/AMB ternary copolymer plugging agents specifically tailored for high-temperature and high-salinity oilfield environments. Through systematic DFT screening of fourteen functional monomer candidates, we identified sodium 3-acrylamido-3-methylbutanoate (AMB) as the optimal third component based on its exceptional electrostatic interactions with AM/AMPS. MD simulations revealed that AMB’s sulfonic acid groups form a high-strength hydrogen bond interaction network that stabilizes the crosslinked structure under extreme conditions. Experimentally synthesized terpolymers were characterized through rheological analysis, FT-IR spectroscopy, scanning electron microscopy (SEM), and thermogravimetric analysis (TGA), with core flooding tests confirming plugging efficiency exceeding 92%. We hope that this work can provide a facile paradigm for cost-effective, computationally guided development of reservoir plugging agents for harsh oilfield environments.

## 2. Experimental Section

### 2.1. Reagents and Instruments

Reagents: Acrylamide (AM, analytical reagent), 2-acrylamido-2-methylpropanesulfonic acid (AMPS, analytical reagent), ammonium persulfate (analytical reagent), 3-acrylamide-3-methylbutyrate (AMB) and MBA (N,N′-methylenebisacrylamide) were purchased from Macklin. Acrylonitrile, 3,3-dimethylacrylic acid, sulfuric acid, petroleum ether, ethyl acetate, anhydrous sodium sulfate, and absorbent cotton were obtained from Aladdin Reagent Co., Ltd. (Shanghai, China).

Instruments: Three-necked flask, separatory funnel, pipette gun (DLAB Scientific Inc., Beijing, China), electronic balance (METTLER TOLEDO, Zurich, Switzerland, 0.1 mg precision), magnetic stirrer (IKA, Staufen, Germany), constant temperature water bath (Julabo, Seelbach, Germany), rotary evaporator (BUCHI R-300, Flawil, Switzerland), vacuum drying oven (BINDER VD53, Tuttlingen, Germany), and nitrogen protection device.

### 2.2. Theoretical and Experimental Methods Employed

#### 2.2.1. Computational Methods for DFT and Molecular Dynamics Simulations

Using GaussView 6.0 software [39], molecular models of 14 candidate monomers were initially constructed. Geometry optimizations were subsequently carried out using Gaussian 16 [40]. All structures were optimized at the B3LYP-D3(BJ)/6-311G(d,p) level of theory [41,42], and the optimized structures with the lowest total energies were selected for subsequent analysis. Molecular electrostatic potential (MEP) calculations were performed at the same theoretical level to evaluate the charge distribution characteristics of the monomers.

Subsequently, a combined system consisting of AM, AMPS, and AMB was constructed and optimized using Gaussian 16. Based on the aforementioned method, the weak interaction energy of the combined system was calculated at the 6-311 + G(d,p) basis set level, with basis set superposition error (BSSE) correction taken into consideration. The interaction energy was calculated according to the following equation: E(inter) = E(AB) + E(BSSE) − E(A) − E(B). All molecular structures were visualized using GaussView 6.0.

To investigate the structural behavior of AM/AMPS/X ternary copolymers under high-temperature (140 °C) and high-salinity reservoir conditions containing divalent cations (Ca^2+^ and Mg^2+^ concentrations of 1.0 × 10^4^ mg/L), molecular dynamics (MD) simulation energy analysis was performed. To simplify the model, an AM-AMPS-AMB molecular chain was constructed with a molar ratio of 7:3:3, while the mass fraction ratio was controlled at 1:1.2:1.1. Structural optimization was carried out using Gaussian 16 with the B3LYP method, combined with D3-BJ dispersion correction and the 6-311G(d,p) basis set. Full-atom molecular dynamics simulations of AM/AMPS and AM/AMPS/AMB polymers were subsequently performed using the GROMACS software package (version 2020.3) [43].

For the AM-AMPS-AMB system simulated at high temperature under conventional conditions, charge equilibration of the AMB monomer was achieved by adding Na^+^ ions. Initially, 25 AM-AMPS-AMB molecules were randomly placed in a cubic simulation box with dimensions of approximately 10 × 10 × 10 nm^3^. Subsequently, 75 Na^+^ ions were added to neutralize the system charge, followed by the addition of sufficient water molecules for simulation at 140 °C.

For the AM-AMPS-AMB system simulated at high temperature under high-salinity conditions, explicit water molecules and ions (Na^+^, Ca^2+^, Mg^2+^, and Cl^−^) were included to reproduce the target salinity environment. Initially, 25 AM-AMPS-AMB molecules were randomly placed in a cubic simulation box with dimensions of approximately 10 × 10 × 10 nm^3^. Subsequently, 275 Na^+^ ions, 25 Mg^2+^ ions, and 25 Ca^2+^ ions were added, followed by the addition of 300 Cl^−^ ions to neutralize the system charge. Finally, sufficient water molecules were added, and the system was simulated at 140 °C.

The specific parameters of the two systems were as follows. All atomic charges were calculated using Gaussian 16 at the B3LYP/6-311G(d,p) theoretical level based on the restrained electrostatic potential (RESP) method. The molecules were described using the GAFF2 force field [44], while water molecules were modeled using the TIP3P model [45]. The cutoff distance for van der Waals and short-range electrostatic interactions was set to 1.2 nm, whereas long-range electrostatic interactions were treated using the Particle Mesh Ewald (PME) method [46]. Before energy minimization and equilibration, the net charge of the entire system was adjusted to zero to ensure the accuracy of electrostatic energy calculations, and the conjugate gradient method was employed for energy minimization. After system construction, the temperature and pressure were controlled using the V-rescale thermostat [47] and the Berendsen barostat [48], respectively. The system was equilibrated for 2 ns under both NVT and NPT conditions with a time step of 1 fs. Subsequently, a 100 ns production simulation was performed under NPT conditions with a time step of 2 fs at 140 °C.

#### 2.2.2. Synthesis of Sodium 3-Acrylamido-3-Methylbutanoate (AMB)

Acrylonitrile (2.73 mL, 0.04 mol), 3,3-dimethylacrylic acid (4 g, 0.04 mol), and distilled water (0.36 mL, 0.02 mol) were mixed in a round-bottom flask and cooled to 0 °C using an ice bath. Concentrated sulfuric acid (4.59 mL, 0.083 mol) was then added dropwise while maintaining the temperature at 0 °C, and the mixture was stirred for 18 h. After the reaction, the mixture was cooled to 0 °C, and 20 mL of distilled water was added to precipitate unreacted starting materials, which were removed via vacuum filtration. The product was extracted using chloroform, and the solvent was removed by rotary evaporation. Subsequently, the product was purified through several recrystallizations using a petroleum ether/methyl ethyl ketone mixture. Finally, residual solvents were removed in a vacuum drying oven to obtain the pure product.

#### 2.2.3. Synthesis of the Copolymers

AM, AMPS, and the crosslinking agent MBA were dissolved in water. Ammonium persulfate initiator was added after adjusting the pH, and the solution was stirred magnetically until homogeneous. The mixture was then sealed and heated at 140 °C for 2 h to complete polymerization. For the preparation of the AM/AMPS/AMB hydrogel, AM, AMPS, MBA, and the functional monomer AMB were dissolved in water at a mass ratio of 7:3:2:3 (AM:AMPS:MBA:AMB). Other operations are the same as above.

#### 2.2.4. Evaluation of Copolymer Properties

Scanning Electron Microscopy (SEM, TESCAN, Brno, Czech Republic): All samples were freeze-dried and sputter-coated with gold for 15 min. Its micro morphology was characterized using scanning electron microscopy.

Fourier Transform Infrared Spectroscopy (FTIR, PerkinElmer, Waltham, MA, USA): The wave number range was 4000 cm^−1^–400 cm^−1^ with a resolution of 0.5 cm^−1^.

Rheological Characterization (Anton Paar GmbH, Graz, Austria): A Physica Anton Paar rheometer was used to test the blocking agent, obtaining its storage modulus and loss modulus. The gap of the parallel plates was set to 1 mm, and the frequency scans were measured from 0.1 to 100 rad s^−1^ at a constant strain of 0.1%.

Thermogravimetric Analysis (TGA, METTLER TOLEDO/TGA2, Zurich, Switzerland): The blocking agent was analyzed using a thermal analyzer under an O_2_ atmosphere from room temperature to 300 °C to measure its weight loss curve with a temperature increase rate of 10 °C min^−1^.

#### 2.2.5. Hydrogel Plugging Performance Test

Solution was introduced into a sand-filling tube and sealed with a bolt. The sealed tube was then placed in an oven at 140 °C for 2 h to allow complete gelation, after which it was cooled to room temperature. Following gelation, the upper interface of the sand-filling tube was connected to the outlet of a parallel-flow pump set at a volumetric flow rate of 3 mL·s^−1^. The pressure was monitored using a high-temperature and high-pressure baffle strength testing device. During the test, the pressure gradually increased until gel sealing failure occurred, characterized by a sudden pressure drop. The instantaneous pressure at the onset of failure was recorded. Subsequently, the pressure increased again to a stable value, which was also documented. Once the gel seal was breached, the hydrogel within the sand-filling tube continued to flow out, and the final stable pressure was recorded. Permeability tests were conducted using a computer-controlled high-temperature, high-pressure core flow apparatus.

## 3. Results and Discussion

The Tahe Oilfield in northwestern China exhibits characteristics of high temperature and high salinity, with differences in reactivity among different functional groups. The high-temperature and high-salinity environment imposes stringent requirements on achieving controllable gelation time and selecting reactive monomers for gel synthesis. Free radical polymerization, due to its mild reaction conditions, diverse monomer options, controllable chain growth mechanism, formation of high molecular weight polymers, flexible reaction types, and controllable crosslinking structure, has become the primary method for constructing polymer network structures. Moreover, its stability and durability further enhance its application potential under harsh conditions. Therefore, considering the environmental responsiveness of chemical groups, the polymer backbone network structure is utilized to establish stable covalent bonds between the polymer and functional monomers through click reactions, thereby forming a high-temperature and high-salinity resistant gel plugging agent with a three-dimensional crosslinked structure.

### 3.1. Determination of the AM/AMPS/X Terpolymer Chain

In this study, 14 functional monomers were selected as additives to improve the temperature resistance of the system. Molecular models of all monomers were constructed using Gaussian 16 software, and the 14 molecular structures were optimized using the B3LYP method based on density functional theory (DFT). The 6-311G(d,p) basis set was employed to optimize the molecular bond lengths, bond angles, and charge distributions (Figure S1).

**Figure 1:**
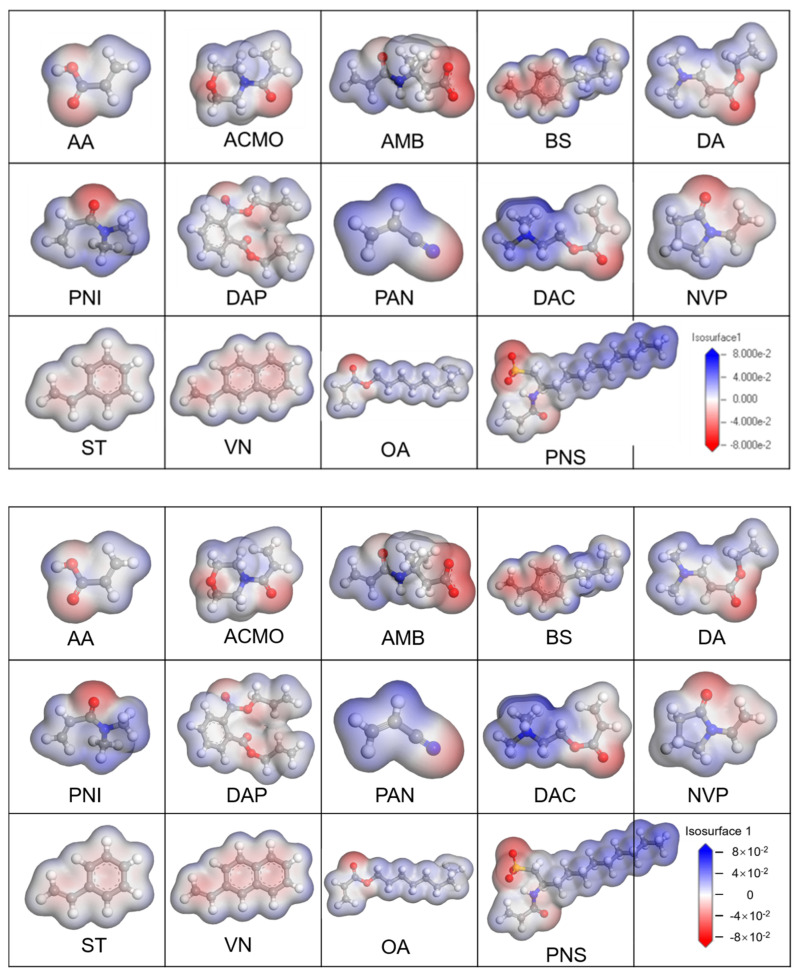
Electrostatic Potential Analysis Diagrams for 14 Functional Monomers.

In addition, an in-depth analysis was conducted by calculating the electrostatic potentials of the 14 optimized monomers. As shown in Figure 1, for most monomers, the negative electrostatic potential regions were mainly concentrated around the oxygen and nitrogen atoms, whereas the positive electrostatic potential regions were primarily distributed around the hydrogen atoms in the hydrocarbon groups. Owing to the negative electrostatic potential associated with most oxygen and nitrogen atoms, these atoms can be regarded as the dominant hydrogen bond acceptors, while the hydrogen atoms can be considered the primary hydrogen bond donors in the molecular structures.

For monomers containing abundant functional groups, such as ACMO, AMB, and DAC, the distributions of negative and positive electrostatic potential regions were more uniform and distinct, providing more potential sites for hydrogen bond formation. In contrast, for monomers containing benzene rings or alkane structures, such as BS, ST, and VN, the electrostatic potential distribution was less pronounced, suggesting that intermolecular interactions in these systems may be mainly dominated by van der Waals interactions, including π–π stacking, with relatively weak electrostatic interactions. Similar to OA and PNS, these monomers possess longer alkyl chains, making them more susceptible to van der Waals interactions with other alkyl chains, which may further reduce their water solubility.

Specifically, owing to the presence of hydrogen atoms in the amino and sulfonic acid groups that are prone to hydrogen bond formation, electrostatic interactions become more favorable when the monomers carry negative charges. As shown in Figure S2, the intermolecular interaction energies (Eint) of the AMPS-AM, AMB-AM, and AMPS-AMB complexes were further compared. The calculated results showed that the Eint value of the AMPS-AMB complex formed through hydrogen bonding was more negative, reaching −8.26 kcal/mol, whereas the Eint value of the AMB-AM complex was only −4.98 kcal/mol. These results indicate that AMB can more readily interact with the other two monomers through hydrogen bonding.

In general, an increase in the number of hydrogen bonds is beneficial for gel formation, and the formation of multiple intermolecular hydrogen bonds is generally conducive to improving the high-temperature stability of materials. Therefore, AM-AMPS-AMB was selected as the research system for further investigation.

### 3.2. Molecular Dynamics Calculations for Different Functional Monomers

To determine the stability of the AM/AMPS/X terpolymer, we conducted full-atomic molecular dynamics simulations. For simplification, we first modeled the polymer chains using a monomer ratio of AM:AMPS:Functional Monomer X = 7:3:3. Using Materials Studio (MS, 2023) software, a series of AM/AMPS/X polymer chains were constructed. To realistically simulate a high-temperature and high-salinity environment (140 °C, 1.0 × 10^4^ mg L^−1^ of Ca^2+^ and Mg^2+^), the quantities of other necessary molecules such as H_2_O, Na^+^, Ca^2+^, Mg^2+^, and Cl^−^ were determined, as shown in Table 1. Using the Forcite module in Materials Studio with the COMPASSIII forcefield, each molecule was optimized for charges. After charge assignment, Ca^2+^, Mg^2+^, Na^+^, and Cl^−^ were assigned their respective charge values. For negatively charged ions AMB (sodium 3-acrylamido-3-methylbutanoate) and PNS (Sodium polystyrene sulfonate), Na^+^ was used for charge balancing. All molecules were assigned charges for each atom using the COMPASSIII forcefield. Energy values were extracted directly from the report file generated after the calculation.

#### 3.2.1. All-Atom Molecular Dynamics Analysis of Am/AMPS and AM/AMPS/AMB Monomers

Counterions (e.g., Na^+^) specifically required to neutralize the net negative charge originating from anionic groups within AMB and PNS monomers were not added beyond those included for salinity balance, resulting in a simulation box with an overall net negative charge. However, due to the extremely high ionic strength (~10^4^ mg L^−1^ total dissolved solids), the resulting electrostatic effects are expected to be strongly screened (Debye length << 1 nm). Therefore, the dominant short-range interactions—particularly the dynamic binding of Ca^2+^ and Mg^2+^ to anionic sites–are accurately captured, and the absence of full charge neutralization does not significantly affect the local conformational behavior of the polymer chains.

#### 3.2.2. All-Atom Molecular Dynamics Analysis of AM/AMPS/X Systems

Based on the MD simulation results, the AM/AMPS/AMB terpolymer demonstrates balanced and favorable performance across key intermolecular forces, including electrostatic interactions, van der Waals forces, and kinetic stability. Molecular dynamics simulations of AM/AMPS with other potential monomers were also conducted.

**Figure 2:**
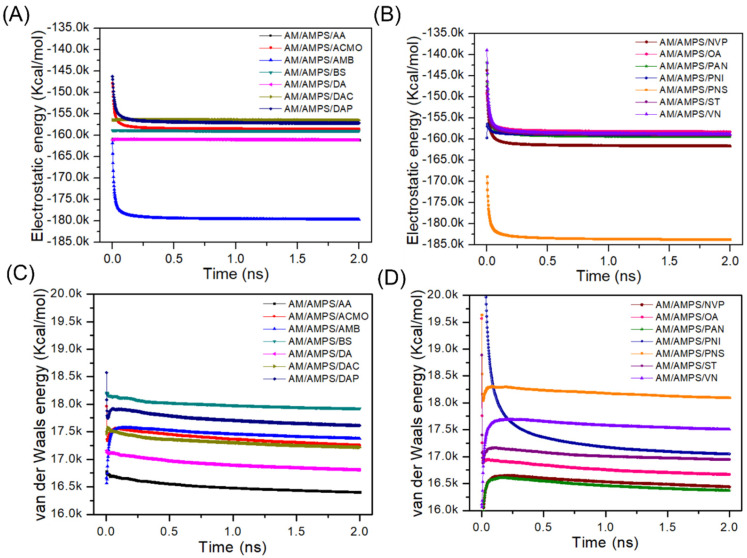
The electrostatic energy (**A**,**B**) and van der Waals energy (**C**,**D**) of the AM/AMPS/X.

As shown in Figure 2A,B, the AM/AMPS/DAP system exhibits the highest electrostatic energy (−135,000 kcal mol^−1^), indicating relatively weak electrostatic associations, likely due to steric hindrance from its benzene ring and ester group. In contrast, the AM/AMPS/AMB and AM/AMPS/PNS systems display the lowest electrostatic energies (−180,000 and −184,000 kcal mol^−1^, respectively), suggesting stronger intermolecular associations that enhance stability under high-temperature and high-salinity conditions. Other systems, such as OA, VN, ST, and PNI, show intermediate values ranging from −155,500 to −165,000 kcal mol^−1^.

Regarding van der Waals interactions (Figure 2C,D), all systems exhibit positive energies with relatively minor differences, confirming that electrostatic forces dominate over van der Waals interactions in these terpolymer systems. Notably, the AM/AMPS/PNI (Figure 2D) system shows an anomalous transition in van der Waals energy, warranting further investigation.

**Figure 3:**
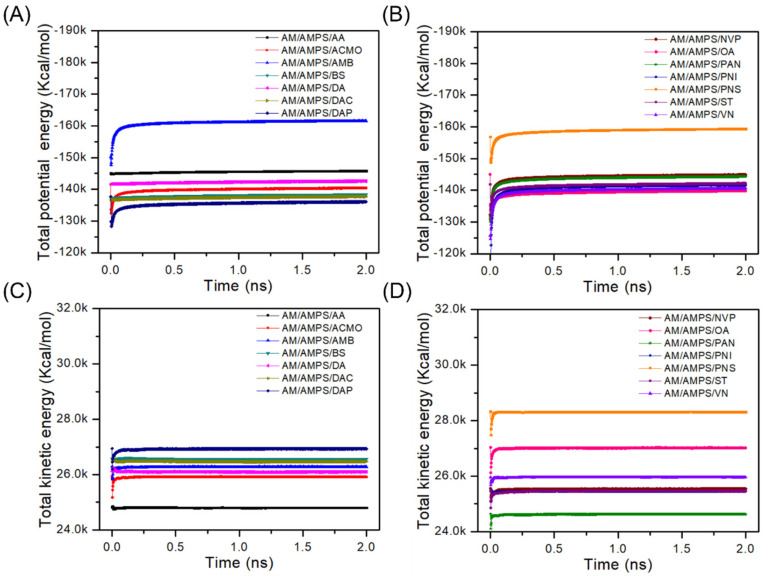
The total potential energy (**A**,**B**) and the total kinetic energy (**C**,**D**) of the AM/AMPS/X.

As depicted in Figure 3A,B, the AM/AMPS/AMB and AM/AMPS/PNS terpolymer systems exhibit the lowest total potential energy, reaching −160,000 kcal mol^−1^. This indicates strong electrostatic energy interactions between AMB and PNS with AM and AMPS. This phenomenon can be attributed to the presence of negatively charged carboxyl and sulfonate groups in AMB and PNS, respectively, which engage in strong interactions with hydrogen bond acceptors like amines. For the AM/AMPS/AA terpolymer system, the total potential energy is relatively low at −145,000 kcal mol^−1^, a difference of 15,000 kcal mol^−1^ compared to the former. This suggests that despite the presence of a carboxyl group capable of electrostatic energy, the interaction with amines is not as strong under high-temperature and high-salinity conditions. Furthermore, the AM/AMPS/ST, AM/AMPS/NVP, and AM/AMPS/PNI terpolymer systems display similar total potential energies, all around −140,000 kcal mol^−1^. This trend aligns with their electrostatic energy profiles, indicating that the phenyl rings and methyl groups in these monomers do not contribute to strong binding interactions compared to negatively charged carboxyl and sulfonate groups.

Regarding the total kinetic energy, the AM/AMPS/PNS terpolymer system exhibits the highest value, reaching 28,000 kcal mol^−1^ at 2 ns, which is approximately 3500 kcal mol^−1^ higher than the lowest energy system, AM/AMPS/PAN. As shown in Figure 3C,D, the AM/AMPS/AA, AM/AMPS/NVP, and AM/AMPS/PNI terpolymer systems possess comparable total kinetic energies, all around 25,500 kcal mol^−1^. Additionally, the AM/AMPS/DAP and AM/AMPS/OA systems also display higher total kinetic energies of 27,000 kcal mol^−1^. The magnitude of the total kinetic energy is closely related to the molecular flexibility; monomers like PNS, OA, and DAP, which possess multiple rotatable and complex structures, tend to exhibit more vigorous molecular motion under high-temperature and high-salinity conditions. Conversely, monomers like AA, NVP, and PAN, with simpler structures and fewer atoms, demonstrate less pronounced molecular motion.

While the van der Waals energies among these 14 terpolymer systems show minor differences, the electrostatic energy, total potential energy, and total kinetic energy exhibit distinct variations, particularly with the AM/AMPS/AMB and AM/AMPS/PNS systems consistently displaying the lowest energies. However, the comparison of total kinetic energies reveals that PNS, due to its multiple flexible and rotatable structures, may experience compromised stability under high-temperature and high-salinity conditions. In summary, based on MD simulations, the AM/AMPS/AMB terpolymer is anticipated to exhibit superior stability under high-temperature and high-salinity environments.

#### 3.2.3. All-Atom Molecular Dynamics Analysis of AM/AMPS and AM/AMPS/AMB Polymers

To further investigate the stability of the AM/AMPS/AMB system under high-temperature and high-salinity conditions, all-atom molecular dynamics (AAMD) simulations were performed to examine its assembly behavior in aqueous solution. To simplify the computational model, the polymer chain was constructed using a monomer ratio of AM:AMPS:AMB = 7:3:3 (mass ratio of 1:1.2:1.1, owing to the similar molecular weights of the three monomers). Initially, 25 AM-AMPS-AMB molecules were randomly distributed in a cubic water box with an initial edge length of 100 Å. To reproduce the high-temperature and high-salinity environment (140 °C, 1.0 × 10^4^ mg/L Ca^2+^ and Mg^2+^), the corresponding numbers of Na^+^, Ca^2+^, Mg^2+^, and Cl^−^ ions were introduced into the system (Table 2 and Table 3). Subsequently, a 100 ns molecular dynamics simulation was carried out under high-temperature and high-salinity conditions.

As shown in Figure 3, multiple AM/AMPS/AMB molecules gradually associated and eventually formed distinct aggregates after 100 ns. The average root-mean-square deviation (RMSD), which was used to evaluate the extent of molecular motion, remained at 4.55 ± 0.12 nm, indicating that the system maintained structural stability throughout the simulation. The average solvent-accessible surface area (SASA) of the system was 300.16 ± 18.01 nm^2^. Notably, the SASA decreased significantly during the initial 0–30 ns period, suggesting reduced solvent exposure and the gradual formation of denser aggregates. Similarly, the radius of gyration (Rg) gradually decreased within the first 40 ns, reflecting the transition of the system from a dispersed state to a more compact aggregated structure.

**Figure 4:**
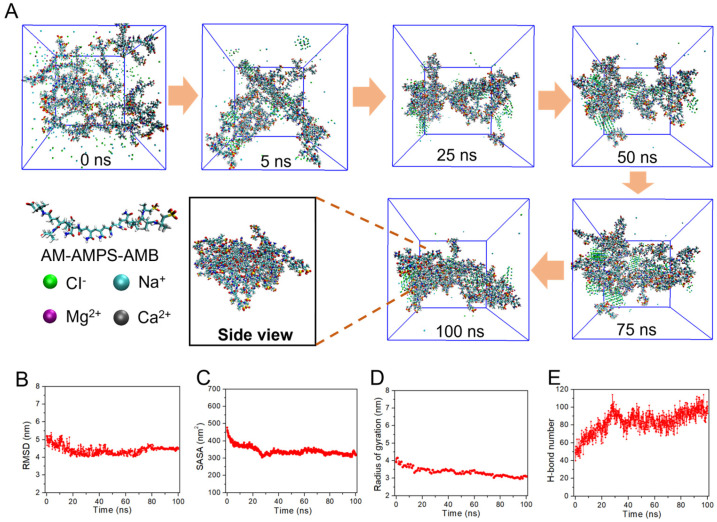
High-temperature molecular dynamics simulations of the AM/AMPS/AMB system under high-temperature and high-salt conditions.

Hydrogen bond analysis over the entire 0–100 ns simulation period revealed that intermolecular hydrogen bonds acted as physical crosslinking sites between the AM/AMPS/AMB molecular chains. Under high-temperature and high-salinity conditions, the RMSD, SASA, and Rg values exhibited only minor fluctuations during the final 50 ns and remained within relatively stable ranges. Moreover, a similar aggregation evolution was observed for the AM/AMPS/AMB system simulated under conventional conditions without the addition of large amounts of Na^+^, Ca^2+^, Mg^2+^, and Cl^−^ ions (Figure 4A–E). These results suggest that the multiple intermolecular hydrogen bonds between the molecular chains play an important role in maintaining the structural stability of the system and suppressing molecular dissociation under harsh conditions.

Notably, hydrogen bond statistics showed that the number of hydrogen bonds under high-temperature and high-salinity conditions was slightly lower than that under conventional conditions, indicating that the presence of large amounts of ions indeed affected the intermolecular interactions within the system. To further elucidate the ion–polymer interactions, the radial distribution functions (RDFs) of Na^+^, Ca^2+^, Mg^2+^, and Cl^−^ around the AM-AMPS-AMB chains were calculated (Figure S3). According to the RDF data, all ions exhibited an initial strong peak at approximately 0.5 nm. Among them, Mg^2+^ displayed the highest peak intensity (31.0), followed by Na^+^ (23.0) and Ca^2+^ (22.0), whereas Cl^−^ showed a substantially lower peak intensity (10.0). These results indicate that the affinity of the cations toward the polymer chains, particularly the sulfonate groups, followed the order Mg^2+^ > Na^+^ ≈ Ca^2+^ >> Cl^−^.

At approximately 1.0 nm, Cl^−^ exhibited a second peak with a higher intensity (25.0) than its first peak, whereas the RDF values of the cations decreased markedly at this distance. This behavior suggests that, owing to electrostatic repulsion, Cl^−^ tends to interact indirectly with the polymer through the hydration layer at relatively longer distances (~1.0 nm). In the range of 1.5–3.5 nm, the RDF values of all ions gradually converged to similar levels (6–13), indicating a more uniform ion distribution at longer distances from the polymer chains. Overall, these results demonstrate that Mg^2+^ most readily accumulates near the polymer surface to form strong coordination interactions, whereas Cl^−^ is preferentially excluded from the vicinity of the polymer chains. These findings provide important insights into ion–polymer interactions and the gel stability of the copolymer system under high-temperature and high-salinity conditions.

### 3.3. Performance Evaluation of AM/AMPS/AMB Terpolymer

#### 3.3.1. Copolymer Morphology

**Figure 5:**
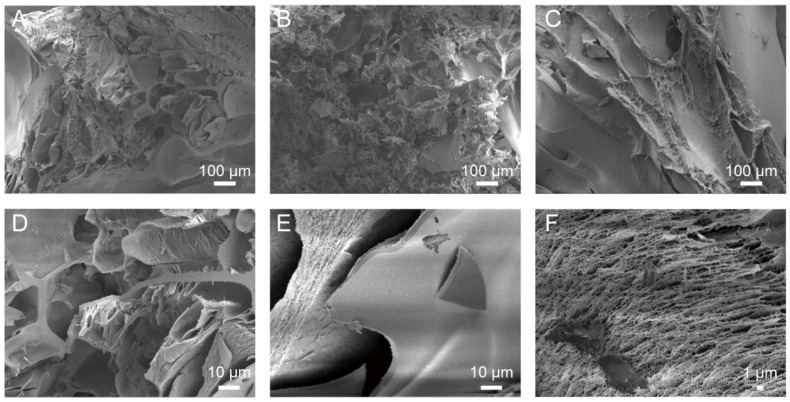
SEM of different size of AM/AMPS/AMB hydrogel (**A**–**C**) low magnification; (**D**–**F**) high magnification, with (**F**) being a partial magnification of (**E**).

Scanning Electron Microscopy (SEM) was employed to investigate the morphology of the AM/AMPS/AMB terpolymer hydrogel. AM/AMPS/AMB hydrogel exhibits layered stacking within its pores (Figure 5C,F). This chemically induced interwoven molecular structure enhances the overall stability of the terpolymer plugging agent. The internal architecture reveals a complex hierarchical structure with high packing density and smaller pores, providing additional mechanical support and chemical stability. Such a hierarchical structure can improve material strength and heat resistance while enabling synergistic stress distribution and reduced swelling across different structural scales. The outer dense layer prevents salt ion penetration, whereas the inner porous structure allows water molecule diffusion, preventing structural collapse. A higher degree of crosslinking in the terpolymer plugging agent can limit deformation at high temperatures and reduce dissolution or swelling under high-salinity conditions. In stark contrast, the binary copolymer gel exhibits only a single lamellar structure in its SEM images (Figure S4), without the multi-layered hierarchical network observed in the terpolymer.

#### 3.3.2. Rheological Testing of Copolymers

**Figure 6:**
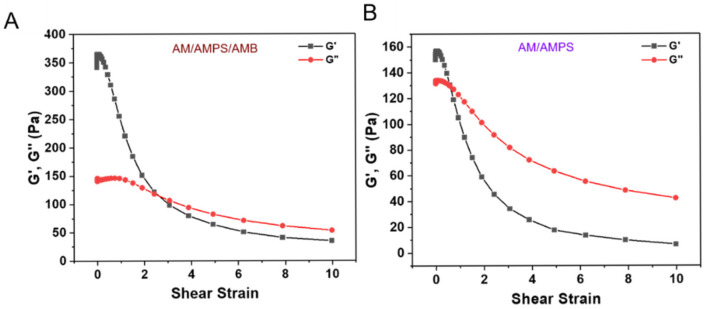
Modulus analysis of AM/AMPS/AMB ternary copolymer (**A**) and AM/AMPS (**B**).

We conducted rheological tests to further investigate the mechanical properties of AM/AMPS binary copolymer gels and AM/AMPS/AMB ternary copolymer hydrogel. As shown in Figure 6A, the AM/AMPS binary copolymer hydrogel exhibits a loss modulus (G’’) ranging from 40 Pa to 140 Pa, decreasing with increasing stress, indicating that the molecular chains are influenced by the applied stress. In contrast, the AM/AMPS/AMB ternary copolymer hydrogel shows a G’’ value between 50 Pa and 150 Pa, which decreases slowly with increasing stress. This suggests that the covalent bonds formed between the polymer chains in the ternary system are more stable than those in the binary system, and the material exhibits hydrogel-like properties [49].

Furthermore, within the entire frequency scanning range, the binary copolymer hydrogel system demonstrates a storage modulus (G’) greater than G’’ at a shear stress of 1, indicating a change in the hydrogel state. As stress increases, the viscosity of the hydrogel increases while its elasticity decreases. For the ternary copolymer system, the crossover point between G’ and G’’ appears at a stress of 3, confirming that the ternary copolymer hydrogel is more stable than the binary copolymer hydrogel.

#### 3.3.3. Characteristics of the Copolymers

**Figure 7:**
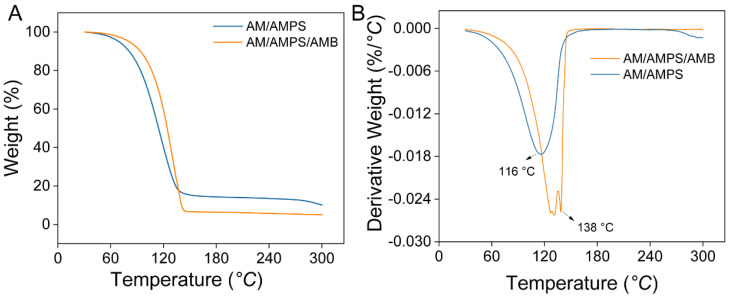
TG (**A**) and DTG (**B**) of AM/AMPS and AM/AMPS/AMB hydrogel.

Furthermore, the prepared AM/AMPS/AMB hydrogel was subjected to high-temperature aging tests to evaluate its long-term structural stability under extreme reservoir conditions. As shown in Figure S3, after being aged in a sealed environment at 150 °C for 48 h, the hydrogel maintained its macroscopic integrity, with no noticeable volumetric shrinkage, dehydration-induced contraction, or structural collapse. Compared with the unaged sample, the color, shape, and elasticity of the hydrogel remained essentially unchanged, indicating that the crosslinked network possesses excellent thermal stability and resistance to dehydration. The aging conditions employed (150 °C, 48 h) significantly exceed the actual reservoir temperature of the Tahe oilfield (140 °C) and the typical operational time frame, serving as an accelerated simulation of long-term stability. TG (Figure 7A) and DTG (Figure 7B) analyses were conducted to evaluate the thermal stability of AM/AMPS and AM/AMPS/AMB hydrogels. Both TG and DTG curves exhibit two distinct weight-loss stages, corresponding to the thermal decomposition of the samples under O_2_. The initial decomposition temperature of the AM/AMPS/AMB hydrogel was observed at 138 °C, which is higher than that of the AM/AMPS hydrogel (116 °C). This improvement is attributed to the enhanced crosslinking density introduced by AMB, which strengthens the polymer network and contributes to its thermal stability. It is speculated that the decomposition of sulfonic acid groups (-SO^3−^) in AMPS occurs at relatively higher temperatures due to their inherent stability. Overall, the incorporation of AMB significantly influences the pyrolysis behavior of the polymer by improving its resistance to thermal degradation [50].

Testing of different hydrogels revealed that the AM/AMPS/AMB hydrogel exhibited the highest plugging efficiency (Table 4), followed by the AM/AMPS/PNS hydrogel, which is consistent with the theoretical simulation results and significantly superior to the other systems.

## 4. Conclusions

In conclusion, this study establishes an integrated computational–experimental framework for the rational design of high-performance oilfield water plugging agents. By combining quantum chemical screening with molecular dynamics simulations, we successfully reduced experimental iterations and accelerated the identification of an optimal ternary copolymer system. Molecular dynamics simulations identified the AMB-containing terpolymer as the most conformationally stable system under coupled high-temperature and high-salinity stress, guiding subsequent experimental validation. Microscopic analysis revealed that AM/AMPS/AMB hydrogels exhibit increased crosslinking density and reduced porosity relative to binary counterparts, consistent with the formation of a denser network architecture driven by strong hydrogen bond interaction between AMB’s sulfonate groups and the AM/AMPS backbone. The resulting AM/AMPS/AMB terpolymer demonstrates superior thermal stability, salt tolerance, and mechanical integrity, positioning it as a promising candidate for water control in extreme reservoir environments. In brief, this work provides a transferable methodology for materials development in oilfield chemistry, demonstrating that computational pre-screening can systematically de-risk experimental synthesis while delivering polymers tailored to specific downhole conditions.

## Figures and Tables

**Table 1:** Simulation conditions for high-temperature and high-salinity environment.

Molecule	Loading	Weight (%)
Poly (AM_3_AMPS_7_X_3_)	5	26.02367
H_2_O	5000	58.80973
Ca^2+^, Mg^2+^	25	0.6542
Na^+^	350	5.25354
Cl^−^	400	9.25887

**Table 2:** Molecular Numbers and Mass Fractions in MD Simulations of AM/AMPS and AM/AMPS/AMB in High-Salinity Systems.

Salt System	(AM/AMPS)		(AM/AMPS/AMB)	
	Molecular Number	Mass Fraction%	Number of Species	Mass Fraction%
poly	14	4.51606	10	4.707589
Ca	77	0.887779	77	0.888603
Mg	127	0.887989	127	0.888813
Cl	1740	17.7449	1740	17.76137
Na	1332	8.809424	1332	8.817598
H_2_O	12957	67.15385	12903	66.93603

**Table 3:** Molecular Numbers and Mass Fractions in MD Simulations of AM/AMPS and AM/AMPS/AMB in water system.

Water System	AM/AMPS		AM/AMPS/AMB	
	Molecular Number	Mass Fraction%	Number of Species	Mass Fraction%
poly	14	5.657628	10	94.34237
H_2_O	14530	5.765165	14833	94.23483

**Table 4:** Pressure result of blocking agent.

Samples	Before Plugging (10^−3^ µm^2^)	After Plugging (10^−3^ µm^2^)	Blocking Rate (%)
AM-AMPS-AMB	8870	708	92.0
AM-AMPS-PNS	5300	650	87.7
AM-AMPS-PNI	2450	360	85.3
AM-AMPS-OA	760	668	12.1
AM-AMPS-AA	870	720	17.2

## Data Availability

The original contributions presented in this study are included in the article/Supplementary Material. Further inquiries can be directed to the corresponding author(s).

## Supplementary Materials

The following supporting information can be downloaded at: https://www.mdpi.com/article/10.3390/polym18111393/s1, Figure S1. Optimization process diagram of six monomers; Figure S2. The intermolecular interaction energies of AMPS-AM, AMB-AM, and AMPS-AMB complexes formed by hydrogen bonding. Figure S3. Radial distribution plots of various ions and AM-AMPS-AMB. Figure S4. SEM images of AM/AMPS hydrogel. Figure S5. Pictures of AM/AMPS/AMB hydrogel after reaction at 150 °C for 3 days. References [51,52] are cited in the supplementary materials.
