# Supplementary material for: Structural Stability of AM/AMPS/AMB Terpolymers Under Simulated Extreme Oilfield Conditions
Source: Polymers (Basel). 2026 Jun 4;18(11):1393. doi: 10.3390/polym18111393 (PMC13259364; doi:10.3390/polym18111393)
Supplement: Supplementary file 1 [file polymers-18-01393-s001.zip › polymers-4192575-supplementary.pdf]

Structural Stability of AM/AMPS/AMB Terpolymers under Simulated Extreme Oilfield Conditions

Peng Xue, Jingxing Wang, Junwei Fang* Qingjie Ma, , Zhi Kang, Linghui Xi, Xiumin Dong, Yi Zhang, Zuguo Yang, and Long He*

All-atom molecular dynamics simulations were performed using GROMACS 2020.3. The GAFF2 force field was employed, and water molecules were modeled using the TIP3P model. A cutoff method was used to handle van der Waals and short-range Coulomb interactions, with a cutoff distance of 1.2 nm. Long-range Coulomb interactions were calculated using the Particle Mesh Ewald (PME) method. Each system was constructed in an 8 nm × 8 nm × 8 nm box using Packmol software. Energy minimization was carried out using the conjugate gradient method. After system construction, temperature and pressure were controlled using the V-rescale thermostat and Berendsen barostat, respectively. The systems were fully equilibrated under NVT and NPT conditions, each for 2 ns with a time step of 1 fs. Subsequently, a 200 ns production simulation was conducted under NPT conditions with a time step of 2 fs. Atomic charges were calculated following optimization to no imaginary frequencies at the BLYP/6-311G(d,p) level of theory with DFT-D3 dispersion correction using Gaussian 16. Charges were then calculated at the B3LYP/def2TZVP level of theory, and RESP charges were derived using the Multiwfn program.

Notes:

1. All data are provided in .xvg files. "salt" indicates a high-salt environment, while "nosalt" indicates a salt-free environment. These files can be opened with a text editor (e.g., Notepad).
2. The attachment is a folder. Taking energy.xvg as an example, data illustration is shown in the figure below. The sum of the two LJ terms represents the van der Waals energy.
3. The following content briefly analyzes the data. It is sufficient to consider the first 100 ns (i.e., 1,000,000 ps), as some systems were only simulated for 100 ns.
4. The folder also contains images for each system at 0, 25, 50, 75, and 100 ns. Files ending in .bmp can be opened directly. .dat files can be processed using tools like Tachyon to achieve the desired resolution.

```
@    xaxis  label "Time (ps)"
@    yaxis  label "(kJ/mol)"
@TYPE xy
@ view 0.15, 0.15, 0.75, 0.85
@ legend on
@ legend box on
@ legend loctype view
@ legend 0.78, 0.8
@ legend length 2
@ s0 legend "LJ-14"
@ s1 legend "LJ (SR)"
@ s2 legend "Potential"
@ s3 legend "Total Energy"
    0.000000  1455.097412  187590.296875  -2091498.500000  -1932905.875000
    2.000000  1471.166260  187666.734375  -2088420.625000  -1929354.750000
    4.000000  1465.508301  189071.828125  -2087866.500000  -1928022.125000
    6.000000  1394.384277  190316.296875  -2091998.875000  -1931397.500000
    8.000000  1508.566650  188613.531250  -2091290.625000  -1931955.750000
```

Horizontal axis unit

Vertical axis unit

The first column is the horizontal axis, and the subsequent columns follow the labels above in the same order.

Brief Data Analysis:

Quality control is shown in the table below. By ensuring consistent mass within the systems, meaningful comparisons between different systems are guaranteed. Ion concentrations were set based on (high salinity 20×10^4 mg/L, high calcium-magnesium ions 1.0×10^4 mg/L).

High Salt System	AM/AMPS Molecule Count	AM/AMPS Mass Fraction %	AM/AMPS/AMB Molecule Count	AM/AMPS/AMB Mass Fraction %
poly	14	4.51606	10	4.707589
Ca	77	0.887779	77	0.888603
Mg	127	0.887989	127	0.888813
Cl	1740	17.7449	1740	17.76137
Na	1332	8.809424	1332	8.817598
H_2O	12957	67.15385	12903	66.93603

Water system	AM/AMPS Molecule Count	AM/AMPS Mass Fraction %	AM/AMPS/AMB Molecule Count	AM/AMPS/AMB Mass Fraction %
poly	14	5.657628	10	94.34237
H2O	14530	5.765165	14833	94.23483

Note: The mass fractions for the AM/AMPS/AMB water system in the original document sum to over 100% (94.34% + 94.23% = 188.57%). This is likely an error. The translation preserves the original numbers but adds this note for clarification, suggesting the user verifies the intended values.

Electrostatic Potential Energy: As shown below, the electrostatic energy decreases significantly in the aqueous solvent after AMB modification, while both systems exhibit similar behavior in the high-salt solution. Furthermore, the electrostatic potential energy of the system is lower in the highly polar high-salt environment, indicating that the system is well-suited for high-salt conditions.

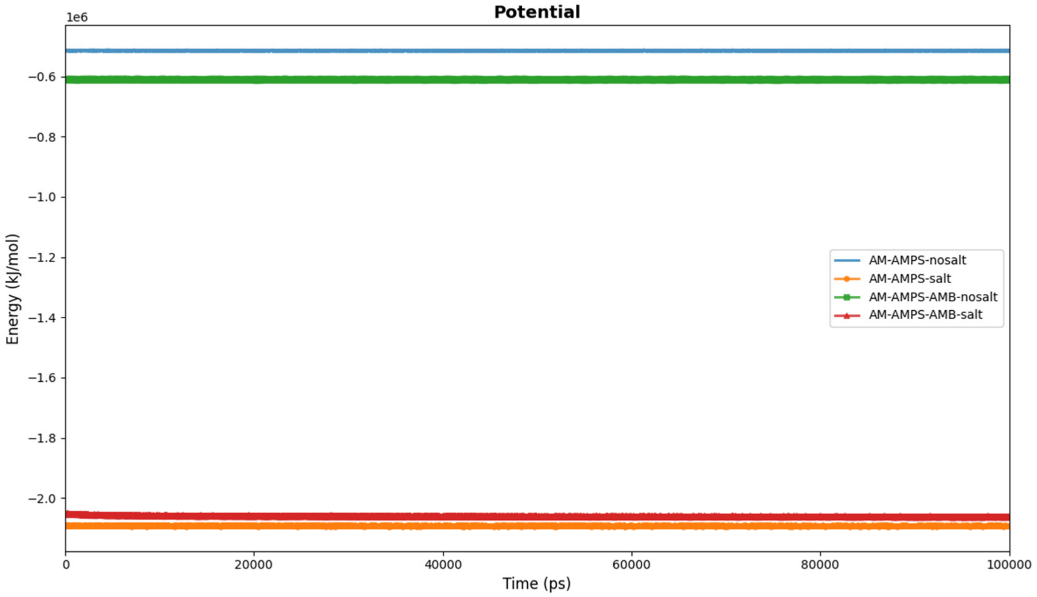

Van der Waals Energy: The results below largely validate the conclusions drawn from the electrostatic potential, suggesting that the primary energy differences in this system arise from van der Waals interactions.

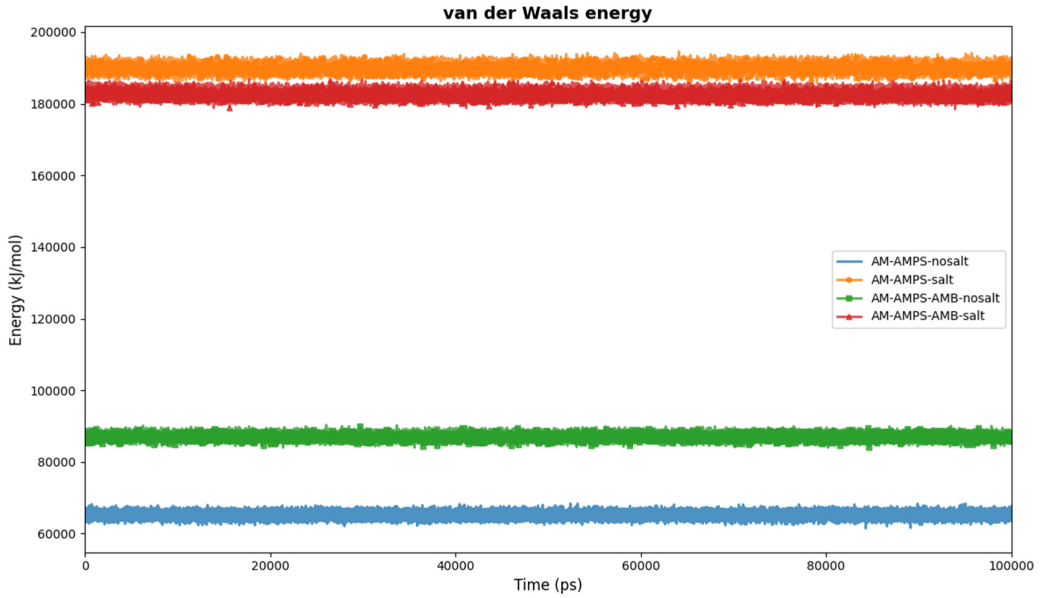

Total Energy: The total energy trend, shown below, is similar to that of the electrostatic potential, indicating that kinetic energy contributions during the thermal bath do not significantly affect the main conclusions.

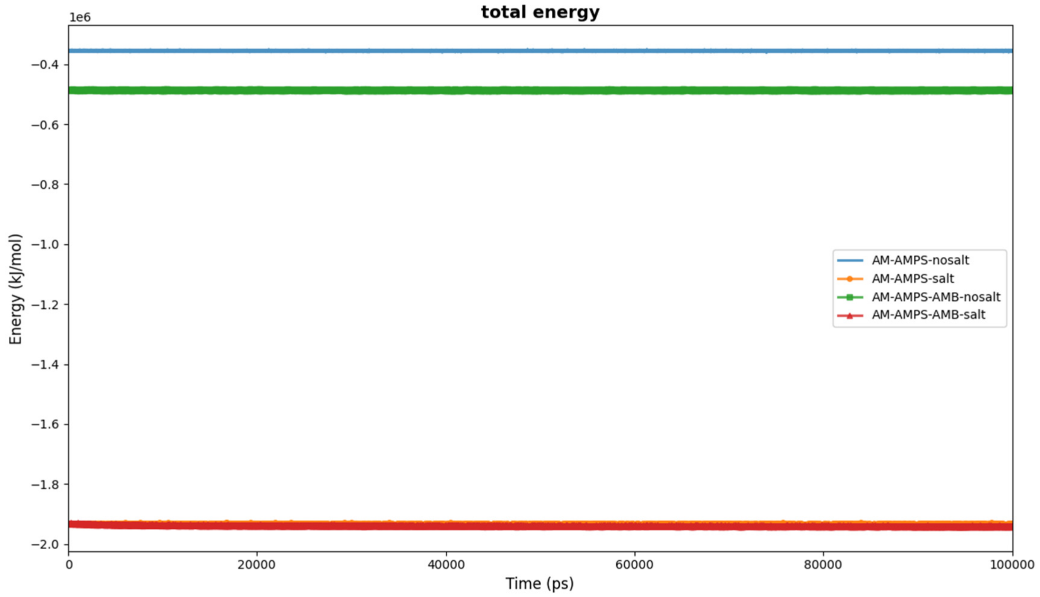

Solvent-Accessible Surface Area (SASA): This indicator reflects the aggregation tendency of molecules during stabilization. In both normal and salt-free environments, the initial simulation stages show similar SASA values with and without AMB modification. However, systems with AMB modification exhibit a significant

decrease in SASA during subsequent simulations, suggesting a higher adhesive/gelling capacity compared to the unmodified version.

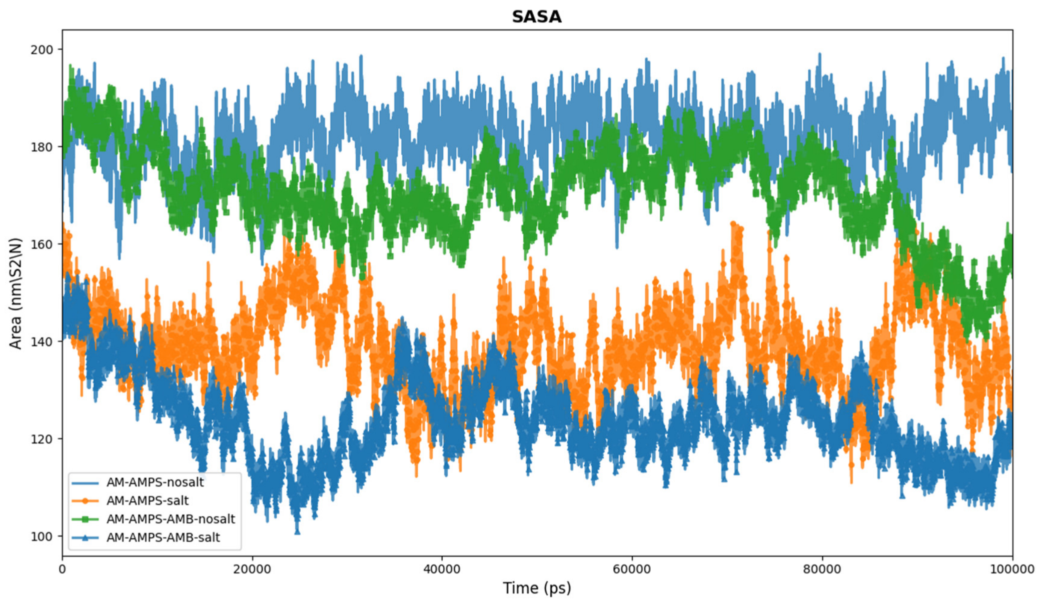

Hydrogen Bond Number: The number of hydrogen bonds is significantly higher in AMB-modified systems compared to unmodified ones. Additionally, hydrogen bond counts are higher in high-salt environments than in ordinary solutions. This indicates the stability of the molecule.

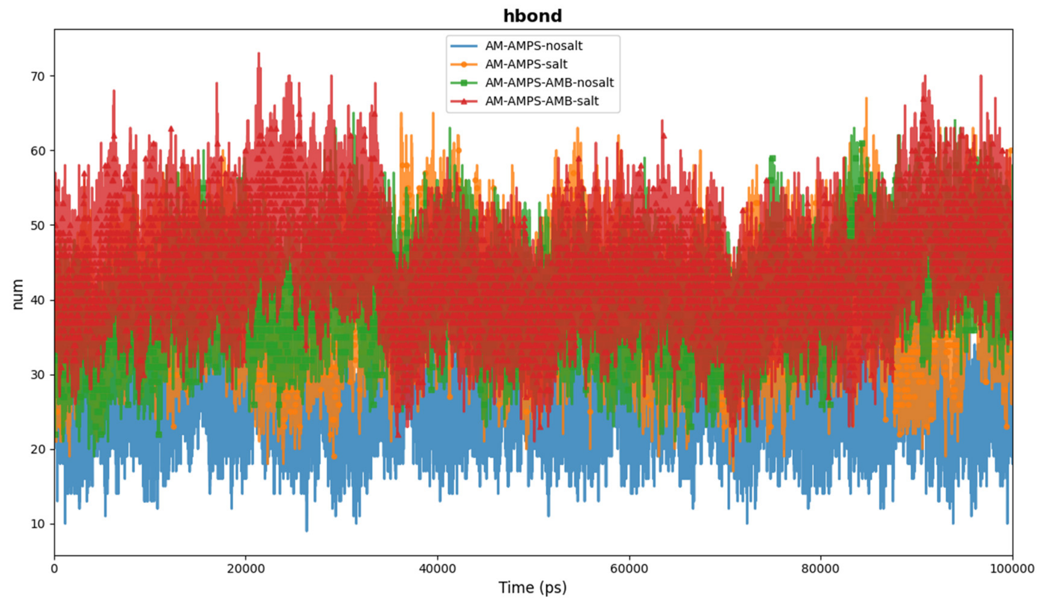

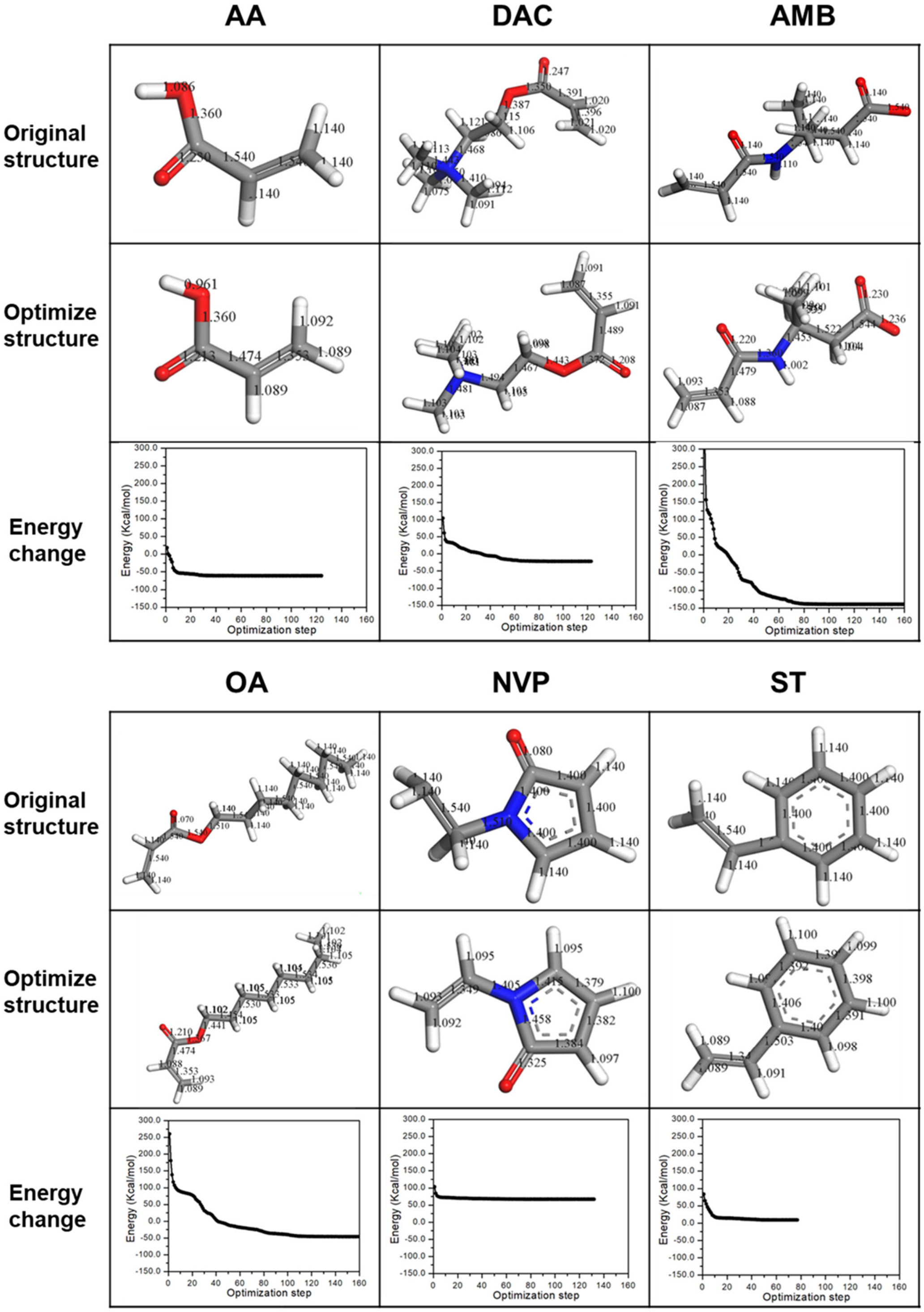

Figure S1 Optimization process diagram of six monomers.

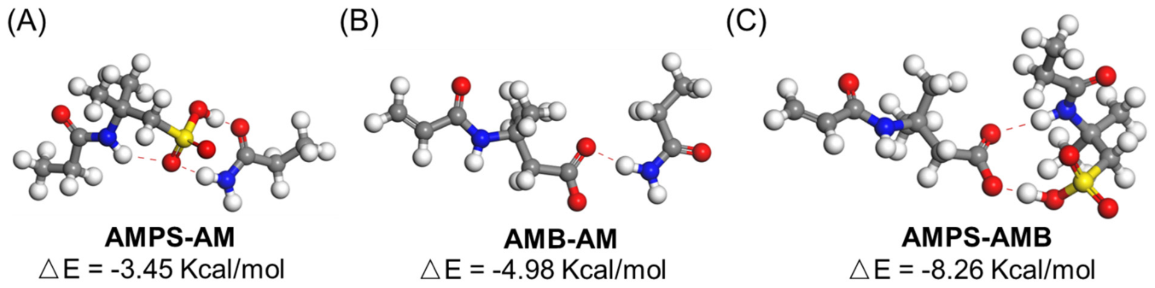

Figure S2. The intermolecular interaction energies of AMPS-AM, AMB-AM, and AMPS-AMB complexes formed by hydrogen bonding.

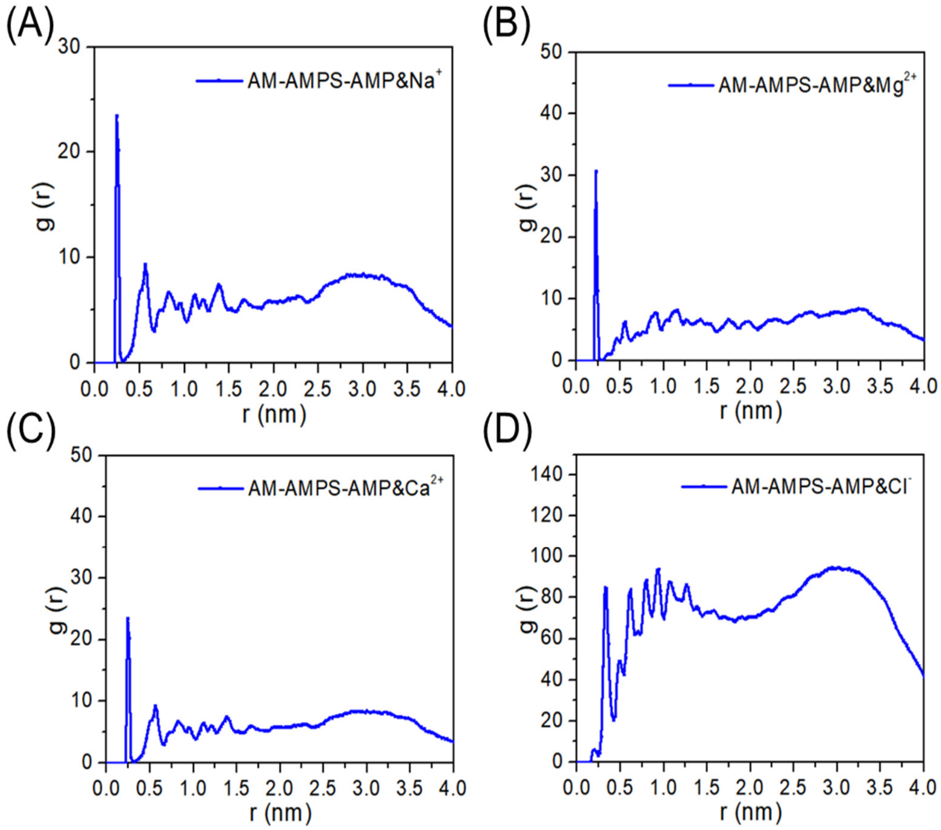

Figure S3. Radial distribution plots of various ions and AM-AMPS-AMB.

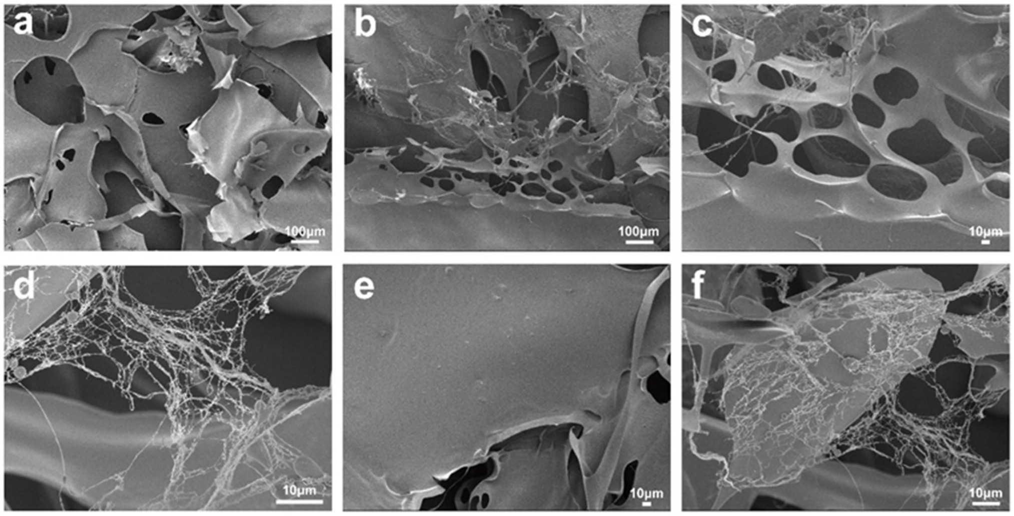

Figure S4. SEM images of AM/AMPS hydrogel.

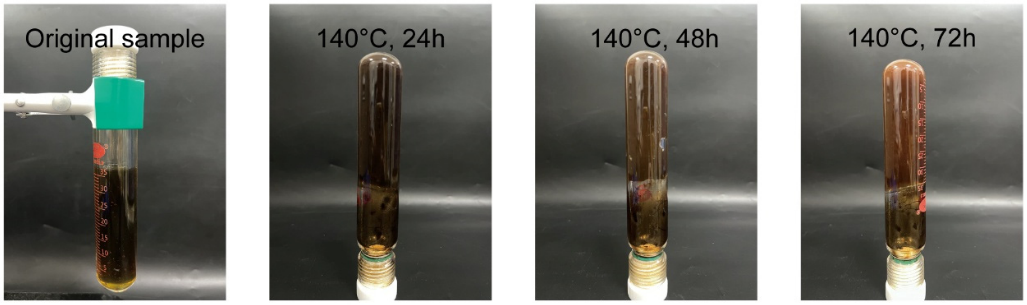

Figure S5. Pictures of AM/AMPS/AMB hydrogel after reaction at 150 ℃ for 3 days.

References:

The following two references are for the Multiwfn software:

- Tian Lu, Feiwu Chen, Multiwfn: A Multifunctional Wavefunction Analyzer, *J. Comput. Chem.* **33**, 580-592 (2012) DOI: 10.1002/jcc.22885
- Tian Lu, A comprehensive electron wavefunction analysis toolbox for chemists, Multiwfn, *J. Chem. Phys.*, **161**, 082503 (2024) DOI: 10.1063/5.0216272
